# Complicated embolisation of late endoleak via direct sac puncture: not all endoleaks are a type II endoleak

**DOI:** 10.1186/s42155-021-00237-3

**Published:** 2021-06-11

**Authors:** Aizat Drahman, Diederick Willem De Boo, Barry Springthorpe, Arvind Deshpande

**Affiliations:** 1 Department of Surgery, Royal Prince Alfred Hospital, Sydney, NSW 2050 Australia; 2 Department of Interventional Radiology, Monash Medical Centre, Melbourne, VIC 3168 Australia; 3 Department of Surgery, John Hunter Hospital, Newcastle, NSW 2305 Australia; 4 Department of Vascular Surgery, John Hunter Hospital, Newcastle, NSW 2305 Australia

## Abstract

**Background:**

Endoleaks after endovascular aortic aneurysm repair (EVAR) occur frequently with type 2 being the most common. Treatment of type 2 endoleaks is indicated if the aneurysmal sac increases in size.

**Case report:**

In this case report, we will discuss a patient who presented with aneurysmal sac size increase 11 years after undergoing EVAR for an asymptomatic abdominal aortic aneurysm which extended into the iliac arteries. Multi-phase CT demonstrated an endoleak with features commonly seen in type 2 endoleaks; pooling of contrast near a lumbar artery orifice on the angiographic phase which increases during the delayed phase. Both internal iliac arteries were sacrificed during the initial EVAR. Percutaneous direct sac puncture was therefor performed and angiogram during the procedure revealed no feeding or draining lumbar arteries. During attempts to embolize the perfused part of the aneurysmal sac non-target embolization into the main body of the graft occurred and the presence of type 3b endoleak was confirmed. The non-target embolization did not result in permanent sequelae.

**Conclusions:**

Type 3b endoleaks are rare and can mimic type 2 endoleaks, which can cause serious complications if not identified properly. Rapid increase in aneurysmal sac size is uncommonly seen in type 2 endoleaks and if present needs to trigger further diagnostic investigations.

## Introduction

Endoleaks after endovascular aortic aneurysm repair (EVAR) occur frequently with type 2 being the most common (Mustafa et al. 2009). The majority are detected during routine follow-up with either computed tomography angiography (CTA), Duplex or Magnetic Resonance Angiography (MRA). The typical distribution of contrast on CTA is usually enough to diagnose what type of endoleak is present. The majority of endoleaks detected are type 2 and these only require an intervention if there is aneurysmal sac expansion. Embolisation can be performed via trans-arterial approach or direct aneurysmal puncture, either transcaval or percutaneous. Trans-arterial approach has reported failure rates as high as 80% and transcaval failure rates vary from 30 to 50%, though this is a relatively new technique with limited publications available (Salvatore et al. 2013). Type 3 endoleak has an estimated incidence of 0.9–3% (Chaikof et al. 2002). It can be further subdivided into type 3a, where there is a modular disruption of the stent graft or component disconnection, or type 3b which is a fabric tear or disintegration of the stent graft (Chang et al. 2013). Type 3 endoleaks, along with type 1 endoleaks, have shown to be the most common cause of rupture and late conversion to open repair post EVAR (Harris et al. 2000). Treatment of type 3 endoleaks differs from type 2 and either involves relining of the stent graft or conversion to open repair. We present a patient who underwent an embolisation via percutaneous direct aneurysmal sac puncture of what was initially thought to be a type II endoleak. During the procedure it however became apparent that it actually was a type 3b endoleak and non-target embolisation occurred.

## Case report

An 82-year old patient presented with increase in aortic aneurysmal sac size increase following an EVAR in 2007 (72-years old at time of EVAR). In 2007 he was treated with a first generation Cook Zenith HLB endo-graft for an asymptomatic infra-renal aortic aneurysm measured 6.0 cm in maximum AP. The right internal iliac artery demonstrated a 5.3 cm aneurysm and the left internal iliac artery measured 1.3 cm. Due to his co-morbidities he was deemed unfit for open aneurysmal repair. He underwent simultaneous bilateral internal iliac artery embolization and EVAR which extended in to both external iliac arteries.

The patient was compliant with annual duplex ultrasound surveillance and had been free of any EVAR-related complications or re-interventions, with a nadir diameter of approximately 6.1 cm to 6.3 cm. In 2018, a routine surveillance with duplex ultrasound demonstrated that the aneurysm sac had grown by 0.5 cm (from 6.3 cm to 6.8 cm) and subsequently in 2019, aneurysm sac had grown to maximum AP diameter of 7.3 cm. The patient had been completely asymptomatic.

**Fig. 1:**
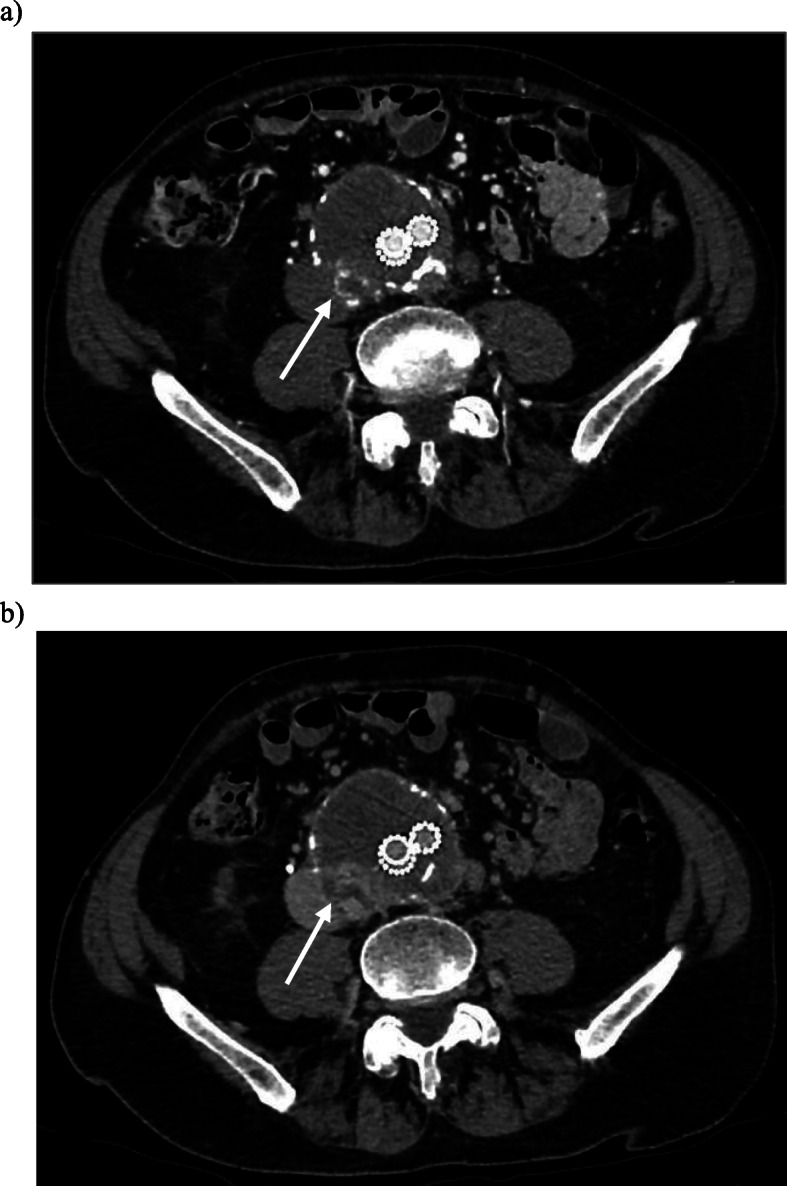
Axial arterial phase CT images of the abdominal aorta demonstrate pooling of contrast (arrow) on the arterial phase (**a**) with increase pooling on the delayed phase (**b**)

**Fig. 2:**
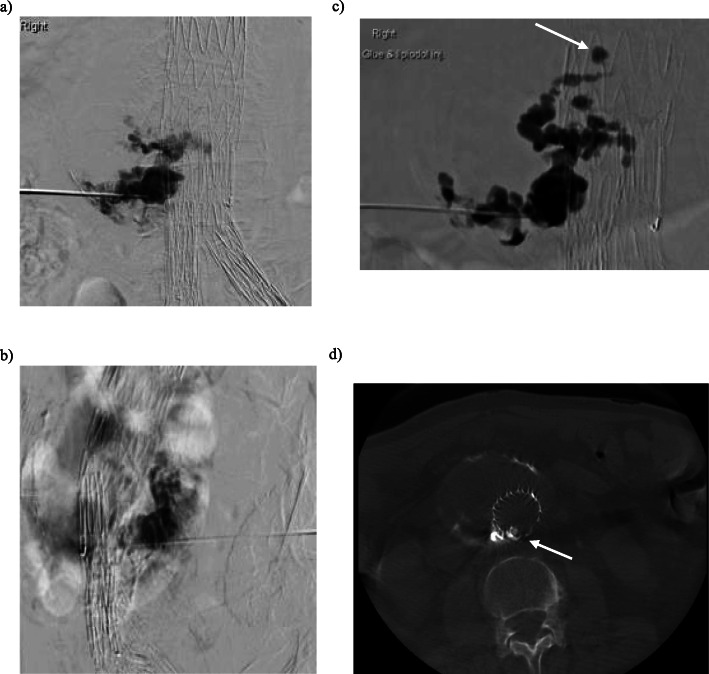
**a/b** Prone PA and lateral digital subtraction angiogram of the abdominal aortic sac injection with iodinated contrast through a 20G access needle demonstrates filling of the aortic sac and no feeding artery. **c** Prone roadmap image of the aortic sac demonstrates filling of the sac with 4:1 Lipiodol/Glue mix (arrow) and penetration through the fabric into the lumen of the graft (arrow). **d** Axial non-contrast cone beam CT (prone) of the abdominal aorta demonstrates non-target embolisation in the body of the stent graft. (arrow)

**Fig. 3:**
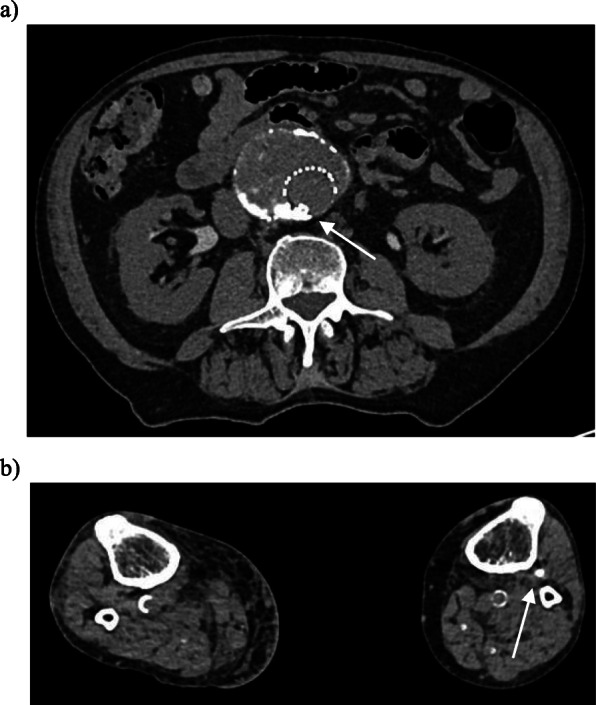
Axial non-contrast CT images of the abdomen and lower limbs performed 1 h after embolisation. Note: contrast excretion is seen from the kidneys due to contrast administered at the time of the angiography procedure. **a** Non-target embolisation of glue is seen (white arrow) in the stent graft lumen (arrow). **b** Axial non-contrast CT at the level of the calf demonstrates non-target embolization within the left anterior tibial artery

At time of current presentation his past medical history included ischaemic heart disease with coronary artery bypass graft in 1993 and pacemaker for cardiomyopathy, atrial fibrillation (on warfarin), dyslipidaemia, hypertension, right peripheral vestibulopathy and previous right cerebellar infarct. The CT with arterial phase and delayed phase was reviewed and demonstrated pooling of contrast on the arterial phase posterior in the aneurysmal sac near the right L4 artery orifice, thought to be the feeding artery (Fig. 1). There is further accumulation of contrast on the delayed phase and the left L4 lumbar artery was presumed to be the draining artery. These imaging findings were interpreted as a type 2 endoleak and given the sac expansion an intervention was indicated based on the guidelines from Society for Vascular Surgery (Zhou 2020). With both internal iliac arteries embolised the decision was made to proceed with percutaneous direct sac puncture and subsequent embolisation. After obtaining informed consent and under antibiotic prophylaxis the patient was put prone on the table and cone-beam computed tomography (CBCT, DynaCT Siemens, Erlingen, Germany) was performed. Using landmarks, the perfused part of the aneurysmal sac was punctured with a 20 G needle via a right translumbar approach. Hand runs during breath holds with iodinated contrast were performed. Multiple angiographic runs in various directions showed a network of channels within the partially thrombosed aneurysmal sac however no feeding or draining arteries, in particular no lumbar arteries, were identified (Fig. 2). Further consultation between the Interventional Radiologist and Vascular Surgeon occurred and in consensus the decision was made to embolise the perfused part of the aneurysmal sac. A mixture of Lipiodol (Guerbet, Villepinte, France) and Hystoacryl (B Braun, Rubi, Spain) with a 4:1 ratio was injected under continuous fluoroscopic (roadmap) control. Opacification of the channels was again observed and only after there was a globule of the embolic material apparent within the stent graft lumen the injection was ceased. Up on further imaging it was noted this globule of embolic material had dislodged (Fig. 2). At this stage the needle was removed and the patient put in the supine position. CBCT confirmed the type 3b endoleak with a small amount of embolic mixture within the stent graft lumen at the site of the fabric tear (Fig. 2). Clinical examination revealed bilateral palpable femoral and popliteal pulses. The left dorsalis pedis as well as right dorsalis pedis and posterior tibial had a strong pulse palpable. The patient did not suffer from motor or sensory deficit. Non-target embolization was confirmed with a non-contrast enhanced and CTA on that same day. The embolic material was noted in the right distal posterior tibial artery and dorsalis pedis, left distal and proximal posterior tibial artery as well as the left popliteal artery (Fig. 3). In the absence of any symptoms, it was decided no further intervention was required and strict 3 monthly surveillance for 12 months with continuation of anti-coagulation. No further sac size increase was observed during initial 6-month follow-up.

## Discussion

Endoleaks are a common finding in follow-up post EVAR occurring in 10–25% of patients (Veith et al. 2002). Type 3 endoleaks are uncommon and 3a, graft module separation, is more common than 3b, graft fabric tear. Our patient developed a delayed type 3b endoleak 12 years post EVAR. Adequately diagnosing a type 3b is challenging on both CTA/MRA as well as digital subtraction angiogram (DSA). The CTA in our case demonstrated findings commonly seen in a type 2 endoleak. Pooling of contrast posterior in the sac near the orifice of the L4 lumbar artery. This increased on the later phases of the CT. Together with the higher incidence of type 2 endoleaks the CTA was interpreted as type 2 and treatment was planned. With both internal iliac arteries embolised prior to the EVAR access to aneurysmal sac was obtained via a direct, translumbar, sac puncture. The angiographic runs once access was established did not demonstrate a feeding nor draining artery which is an unexpected finding in type 2 endoleaks. This was recognised at time of the procedure; however, the findings were not interpreted as type 3b endoleak. In an attempt to embolise the perfused part of the aneurysmal sac embolisation with Lipiodol/Hystoacryl mixture was performed. Only once there was flicker of embolic agent in the lumen of the stent graft was the diagnosis of a type 3b endoleak made. Reviewing the angiographic runs prior to embolisation did not demonstrate the fabric tear, likely because there is rapid dilution of the contrast with non-opacified blood in the aortic stent graft. Fortunately, the non-target embolisation remained asymptomatic on subsequent follow-up. It is likely that more fabric tears are present, however, given the medical background history it was felt that more invasive procedure, whether endovascular or open repair, was not feasible in this patient. The perfused part of the aneurysmal sac as well as the main fabric tear are currently excluded. Patient returned to routine surveillance and has not shown sac growth 6 months post procedure.

In retrospect, the CT demonstrated features commonly seen in type 2 endoleaks; pooling of contrast near a lumbar artery orifice on the angiographic phase which increases during the delayed phase. However, the rate of increase in aneurysmal sac size, 10 mm in 12 months, is unusual for type 2 endoleaks and needs to trigger further work-up. This can be either formal angiogram with or without CBCT and/or contrast enhanced ultrasound if available.

The incidence of Type 3b endoleaks overall is generally low, but early generation stent grafts have been associated with higher rates of endoleaks (Tadros et al. 2014). Cho et al. reported two cases of type 3b endoleak after EVAR with a Cook device. Although not specified for one patient they did not describe the use of a first generation Cook Zenith HLB endo-graft. To date there are no reports of type 3b endoleaks with the HLB endo-graft. Barburoglu et al. reported a Type 3b endoleak 14 months after insertion of an Endurant endograft (Greenberg et al. 2008; Abouliatim et al. 2010). Type 3b endoleak is treated either endovascularly or surgically. Endovascular options include repairing the defect with placement of a new aorto-bi-iliac graft or an aorto-uni-iliac graft with cross-femoral bypass or aortic cuff extension. Explantation, either partial or complete and open repair are the surgical options which carry a high mortality rate of 10% to 40% (Turney et al. 2014).

## Conclusion

In conclusion, type 3b endoleaks are rare and can mimic type 2 endoleaks. Misinterpretation can lead to inadequate treatment with non-target embolisation as occurred in this case. Rapid increase in aneurysmal sac size is uncommonly seen in type 2 endoleaks and if present needs to trigger further diagnostic investigations.

## Data Availability

Data sharing not applicable to this article as no datasets were generated or analysed during the current study.

## Abbreviations

EVAR: Endovascular aortic aneurysm repair
CTA: Computed tomography angiography
MRA: Magnetic Resonance Angiography
CBCT: Cone-beam computed tomography
DSA: Digital subtraction angiogram
